# The Psycho-Affective Roots of Obesity: Results from a French Study in the General Population

**DOI:** 10.3390/nu12102962

**Published:** 2020-09-28

**Authors:** Lena Bourdier, Melina Fatseas, Anne-Solène Maria, Arnaud Carre, Sylvie Berthoz

**Affiliations:** 1 Addictology Department, University Hospital of Bordeaux, 33000 Bordeaux, France; melina.fatseas@u-bordeaux.fr; 2 CNRS, EPHE, INCIA, UMR 5287, Université de Bordeaux, F-33000 Bordeaux, France; sylvie.berthoz-landron@inserm.fr; 3 Private Practice, Stimulus Consulting Ltd., Coastal Road, Cascavelle 90203, Mauritius; annesolenemaria@gmail.com; 4 UVSQ, CESP, INSERM, Université Paris-Sud and Université Paris Descartes, 75014 Paris, France; 5 LIP/PC2S, Grenoble Alpes University and Savoie Mont Blanc University, F-38000 Grenoble, France; arnaud.carre@univ-smb.fr; 6 Department of Psychiatry for Adolescents and Young Adults, Institut Mutualiste Montsouris, 75014 Paris, France

**Keywords:** obesity, food addiction, emotional eating, intuitive eating, depression, anxiety

## Abstract

The aim of the study was to examine the extent to which obese people differ in their emotionally driven and addictive-like eating behaviors from normal-weight and overweight people. A total of 1142 participants were recruited from a general population, by a web-based cross-sectional survey assessing anxiety/depression (Hospital Anxiety and Depression Scale), emotional eating (Emotional Appetite Questionnaire), food addiction (modified Yale Food Addiction Scale), and intuitive eating (Intuitive Eating Scale-2). The statistical design was based on analyses of (co)variance, correlograms, and mediations. A set of Body Mass Index (BMI) group comparisons showed that obese people reported higher levels of depression and emotional eating and that they experienced more severe and frequent food addiction symptoms than overweight and normal-weight people. Associations between anxiety, depression, food addiction symptoms’ count, and the difficulties to rely on hunger and satiety cues were found across all weight classes, suggesting that addictive-like eating may represent a unique phenotype of problematic eating behavior that is not synonymous with high BMI or obesity. Conversely, the interrelation between anxiety/depression, emotional eating, and the difficulties to rely on hunger and satiety cues was found only among obese participants, and negative emotional eating mediated the association between depression and anxiety and the difficulties to rely on hunger and satiety cues. This study emphasizes the necessity to develop more comprehensive approaches integrating emotional dysregulation and addictive-like eating behaviors to improve weight management and quality of life of obese people.

## 1. Introduction

Obesity is a multifactorial disease involving an interplay between environmental, genetic, biological, and psychological factors [1]. Among them, both homeostatic dysregulation, which results in poor interoceptive awareness and low sensitivity to the physiological hunger and satiety signals [2,3,4], and emotional dysregulation [5,6,7] are increasingly being discussed as possible factors involved in non-nutritional eating. Failures in weight management may be partly explained by an incomplete understanding of the psychological obesity risk and maintaining factors [6].

Indeed, while a decrease or suppression of food intake in response to stress and negative mood has been conceived as the natural, typical, distress response because of physiological changes that mimic satiety [8], it is now acknowledged that important individual differences modulate the way people intake food in the same conditions, with as many as 30 to 50% of people who report eating more during stressful periods [9]. Consistently with seminal descriptions of the psychological aspects of hyperphagia and obesity made in the 1950s by Bruch [10], Hamburger [11], and Stunkard [12], and as conceptualized in the Emotionally Driven Eating Model [6,13,14], some individuals appear to be susceptible to unhealthy shifts towards energy-dense and highly palatable (HP) food items when being emotional [15,16,17]. Both experimental and epidemiological studies on this issue have consistently identified overweight and obese people as being particularly prone to these shifts, and these findings are part of the conceptual framework of the recently proposed Clinical Obesity Maintenance Model [6].

From a neurobiological perspective, it is now established that the regulation of food intake originates from the orchestration of the activity of neural circuits involved in both somatic and affective (or emotional) homeostatic processes. These links have been viewed as the basis for the development of undercontrolled, nonhomeostatic, and addictive-like consumption of high-energy-dense and HP foods [18,19]. While there exists no consensual definition of what should be considered addictive-like eating patterns [20], the concept of food addiction (FA) has been recently identified as a potential underlying mechanism of overeating and unsuccessful attempts to reduce calorie intake [21,22]. Such an eating behavior triggers the neurobiological cascade associated with the brain reward pathways, in a similar way as the association between stress (either intrinsic or extrinsic) and drug addiction [19,23,24].

No standardized definition of emotionally driven eating behaviors exists, but the concept of Emotional eating (EE) is generally defined as the overconsumption of food in response to negative effects rather than in response to feelings of hunger, which places the individual at risk for overweight and obesity [9,25]. Emotional eating has been viewed as a potential precursor of compulsive overeating and addictive-like eating behaviors [26,27,28] and accumulating evidence suggests that (i) individuals with high levels of negative affectivity are prone to use food for self-medication purposes and to adopt addictive-like eating behaviors [27,28,29,30], and (ii) that psychological distress has differential effects on anthropometric indices (BMI, waist circumference and weight gain) as a function of the level of EE or FA (i.e., that emotionally driven and addictive-like eating act as mediators between low mood and high body weight) [28,31,32]. In addition, a diagnosis of FA, as measured by the Yale Food Addiction Scales (YFAS, mYFAS, YFAS2.0, mYFAS2.0), has been found to be positively associated with depression and EE, and FA and EE are prevalent among high BMI populations [33,34,35,36]. However, the extent to which these patterns of association are specific to obesity or concerns all weight classes remains largely unexplored.

Different studies have examined emotionally driven and addictive-like eating behaviors in high BMI populations, but the majority of them either included patients seeking bariatric surgery or they did not clearly differentiate obese people from overweight people [33,34]. Of note, besides the prevalence of FA, the question of whether obese and overweight people differ in the type of FA symptoms they endorse has been overlooked. Nonetheless, we suggest that a better understanding of these issues should help to tailor additional therapeutic options.

Accordingly, the aim of the present study was to examine, in a sample from the general population, the extent to which obese people differ in their emotionally driven and addictive-like eating behaviors not only from normal-weight people but also from overweight people. We expected the obese group would present the highest levels of these behaviors and symptoms. Moreover, we expected to observe stronger positive associations between the level of psychological distress and both the EE score and the FA symptoms score and stronger negative associations between the level of psychological distress and the level of sensitivity to the physiological hunger and satiety signals among the obese group than the other two groups.

## 2. Materials and Methods

### 2.1. Participants and Procedures

Participants were recruited from a larger web-based cross-sectional survey data-set on eating behaviors [28]. All participants were adults and engaged freely in the study for no financial compensation. The web survey link was sent to participants using online social media and platforms and via institutional mailing lists. The first page of the online survey included information regarding the purposes of the study and a note about the fundamental principles of ethical scientific research and the French Code of Ethics of Psychologists. Information about anonymity, confidentiality, and data protection was given. In addition, it was explained that all the provided and collected information would only be used to meet the objectives of the research. Participants were then asked to provide their electronic, informed consent prior to their participation in the study. The survey demanded between 25 and 30 min to complete. For the present study, we included participants with a BMI of at least 18.5 kg/m^2^ and with no missing data for our variables of interest, reducing the initial sample size from 1349 participants to 1142 participants.

### 2.2. Ethical Considerations

This study was conducted in accordance with the ethical standards described in the Declaration of Helsinki. The study was approved by the Ethics Committee of the University Savoie Mont Blanc (CEREUS_2016_4).

### 2.3. Measurements

Self-reported sociodemographic information was collected (age, gender, and level of education). Participants also provided self-reported height and weight to calculate Body Mass Index (BMI) as weight (kg)/height (m)^2^. Standard categories of BMI were constituted according to the World Health Organization: 18.5–24.9 (normal-weight), 25–29.9 (overweight), and 30 or more (obesity).

#### 2.3.1. Anxiety and Depression

The Hospital Anxiety and Depression Scale (*HAD*) is a 14-item self-report questionnaire that assesses the level of anxious and depressive symptoms during the past week [37,38]. The HAD includes two subscales: Anxiety (7 items) and Depression (7 items). Participants were asked to rate the extent to which they agreed with each statement on a 4-point scale rating from 0 to 3. In this study, Cronbach’s alphas for the *HAD Anxiety* and *Depression* subscales were 0.79 and 0.75, respectively.

#### 2.3.2. Emotional Eating

The Emotional Appetite Questionnaire (*EMAQ*) is a 22-item self-report questionnaire assessing variations of food intake in response to different emotional states and situations [39,40]. The scale contains 9 items assessing negative emotions, 5 items assessing positive emotions, 5 items assessing negative situations, and 3 items assessing positive situations. For each item, participants were asked to rate on a 9-point Likert-type scale whether they ate less (from 1 to 4), the same (5), or more (from 6 to 9) food compared to usual. In the present study, we used the EMAQ global positive score (obtained by averaging the EMAQ-positive emotions and positive situations scores) and the EMAQ global negative score (obtained by averaging negative emotions and negative situations scores). In this sample, Cronbach’s alphas were 0.88 for the *EMAQ-Positive* subscale and 0.83 for the *EMAQ-Negative* subscale.

#### 2.3.3. Intuitive Eating

The Intuitive Eating Scale-2 (*IES-2*) is a self-report questionnaire designed to assess attitudes and behaviors towards eating in response to physiological cues [41,42]. The IES-2 encompasses 18 items divided into three subscales: *Eating for Physical rather than Emotional Reasons* (EPR: 8 items), *Reliance on Hunger and Satiety Cues* (RHSC: 4 items), and *Unconditional Permission to Eat* (UPE: 6 items). Items were answered using a 5-point response format ranging from 1 (“*Strongly disagree*”) to 5 (“*Strongly agree*”). In this sample, Cronbach’s alpha was 0.90 for the EPR subscale, 0.87 for the RHSC subscale. and 0.70 for the UPE subscale.

#### 2.3.4. Food Addiction

The modified Yale Food Addiction Scale (*mYFAS*) is a short version of the original YFAS [43,44] designed to assess the behavioral indices of addictive-like eating [45]. This 9-item self-report questionnaire was developed for epidemiologic studies. Seven items are based on DSM-IV-TR symptoms of addiction: *Loss of control* (substance taken in larger amount and for a longer period than intended); *Cut down* (persistent desire or repeated unsuccessful attempt to quit); *Time spent* (much time/activity to obtain, use, recover); *Impact activities* (important social, occupational, or recreational activities given up or reduced); *Withdrawal* (characteristic withdrawal symptoms; substance taken to relieve withdrawal); *Despite problems* (use continues despite knowledge of adverse consequences) and *Tolerance* (marked increase in amount; marked decrease in effect). Two additional items (*Clinical distress* and *Clinical impairments*) are used to assess *Clinical significance*. This questionnaire includes five frequency response options that range from 0 (“*Never*”) to 4 (“*More than 4 times/week*”). The mYFAS provides two scoring options: a “Symptom Count” scoring option (i.e., a count of food addiction symptoms, ranging from 0 to 7) and a “Diagnostic” scoring option (presence of 3 or more symptoms in addition to the presence of *Clinical significance*) [45]. In addition, a severity score above the cut-off was calculated for each item of the mYFAS. In this sample, Cronbach’s alpha for the mYFAS was 0.73.

### 2.4. Statistical Analyses

Descriptive statistics were computed using means, standard deviations (SD), and ranges for continuous variables, and using counts and percentages for categorical variables. The main effects of BMI groups for age and gender were tested using one-way analysis of variance (ANOVAs) and Chi-square tests (χ^2^), respectively. Age differed significantly between the three BMI groups. As is known to affect BMI, EE, and FA [46,47], the main effects of BMI groups and comparisons between pairs of BMI groups for the mood and eating variables were performed using separate analyses of covariance (ANCOVAs) with age as the covariate. Effect sizes were estimated using partial eta-squares (η*_p_*^2^) and Cramers’ V. Value of η*_p_*^2^ around 0.01 was associated with a small effect, value around 0.06 was associated with a medium effect, and value around 0.14 was associated with a large effect [48]. A value of Cramer’s V can be interpreted as negligible (0–0.10), weak (0.10–0.20), moderate (0.20–0.30), relatively strong (0.40–0.60), strong (0.60–0.80), or very strong (0.80–1) [49].

To examine if the associations between the mood and eating variables vary by a group of BMI, correlation matrix using Spearman correlation coefficients, and corresponding correlograms were performed in each BMI group separately. A correlogram is a graphical representation of the correlations for all pairs of variables. The color legend of the correlogram shows the correlation coefficients and the corresponding colors [50]. The intensity of the color is proportional to the correlation coefficient (*r*), so strong correlations (i.e., the closest to −1 or 1) are displayed in dark boxes. No significant correlations are displayed in white, positive correlations are displayed in blue and negative correlations are displayed in red.

Finally, based on the finding among the obese group that the level of depression or anxiety, negative emotional eating, and capacity to rely on internal cues to regulate food intake were interrelated, we examined if negative emotional eating (*EMAQ Negative* score) mediated the association between the level of psychological distress (*HAD Depression* or *Anxiety* score) and the reliance on internal cues (*IES-2 Reliance on Hunger and Satiety Cues* score) (see Supplementary Figure S1). We followed the basic steps for mediation analysis [51]:

- Step 1: To show that the predictor was significantly associated with the outcome variable, we estimated the unmediated effects of the *HAD Depression* and *HAD Anxiety* scores on the *IES-2 Reliance on Hunger and Satiety Cues* score (i.e., total effect);
- Step 2: To verify that the predictor was associated with the mediator, we estimated the direct effects of the *HAD Depression* and *HAD Anxiety* scores on the *EMAQ Negative* score (i.e., *a* paths),
- Step 3: To verify that the mediator was associated with the outcome, we estimated the direct effect of the *EMAQ Negative* score on the *IES-2 Reliance on Hunger and Satiety Cues* score (i.e., *b* paths);
- Step 4: To establish that the mediator affects the predictor–outcome relationship, we estimated the direct effects (i.e., *c* paths, adjusted for the mediator) and indirect effects (i.e., *a* × *b* paths) of the *HAD Depression* and *HAD Anxiety* scores on the *IES-2 Reliance on Hunger and Satiety Cues* score.

We used the bootstrapping resampling technique (with a 1000 sample) and reported the estimates (B) and their respective standard errors and confidence intervals as well as the percentage of mediation. 

Analyses of variance, covariance, χ^2^ tests, and mediation models were performed using Jamovi version 1.1, Jamovi, Sydney, Australia [52]. The correlograms were carried out using R 2.15.2, R Core Team, Vienna, Austria [53]. An alpha of 0.05 was retained as a significant threshold for all statistical tests.

## 3. Results

### 3.1. Descriptive Statistics of the Sample

Participant characteristics and scale scores are presented in Table 1. Of the 1142 participants, based on their BMI, 82.1% of them (*n* = 938) reported being normal-weight (NW), 12.9% of them (*n* = 147) reported being overweight (OW) and 5% of them (*n* = 57) reported being obese (OB). Among the obese participants, 63.2% reported moderate obesity (Class 1: BMI of 30 to 34.9), 26.3% reported severe obesity (Class 2: BMI of 35 to 39.9), and 10.5% reported morbid obesity (Class 3: BMI of 40 or higher). The mean ages were 22.7 years (±6.6) for normal-weight participants (75.6% women), 25.3 years (±10.1) for overweight participants (68.7% women), and 28.6 years (±10.3) for obese participants (80.7% women).

### 3.2. BMI Group Comparisons

The main effect of gender was not significant (χ^2^ (2, *n* = 1142) = 4.3; *p* = 0.119). The results showed a main effect for age (F(2,1139) = 23.0; *p <* 0.001; η^2^ = 0.04), and the post-hoc tests (Bonferroni-corrected) highlighted that obese participants were older than the overweight participants, who were themselves older than the normal-weight participants (respectively: OB/OW mean difference = 3.25, SD = 1.2, *p <* 0.05; OB/NW mean difference = 5.86, SD = 1.0, *p <* 0.001; OW/NW mean difference = 2.60, SD = 0.7, *p <* 0.001). In view of this result, all the remaining BMI group comparisons were adjusted for age (ANCOVAs). Table 2 summarizes the BMI group comparisons for the mood and eating behaviors variables.

Concerning mood measures (HAD), there was a main effect for *Depression* (*p <* 0.001) and pairwise comparisons adjusted for age showed that scores were significantly higher among the obese group than the overweight and normal-weight groups (OB/OW mean difference = 1.61, SD = 0.5; OB/NW mean difference = 1.96, SD = 0.4). There was no main effect for *Anxiety* (*p* = 0.220).

The analyses indicated a main effect for positive emotional eating (*EMAQ Positive*: *p <* 0.001) and pairwise comparisons adjusted for age showed that the obese and overweight participants reported lower scores than the normal-weight participants did (OB/NW mean difference = 0.50, SD = 0.1; OW/NW mean difference = 0.32, SD = 0.8). There was also a main effect for negative emotional eating (*EMAQ Negative; p <* 0.001), and pairwise comparisons adjusted for age indicated that the obese participants reported higher scores than the overweight participants, who themselves reported higher scores than the normal-weight participants (OB/OW mean difference = 0.63, SD = 0.2; OB/NW mean difference = 1.20, SD = 0.2; OW/NW mean difference = 0.58, SD = 0.1).

Regarding intuitive eating (*IES-2*), the main effect of BMI groups emerged for the *Eating for physical rather than emotional reasons* subscale (*EPR*: *p <* 0.001) and the *Reliance on Hunger and Satiety Cues* subscale (*RHSC*: *p <* 0.001). Pairwise comparisons adjusted for age highlighted that the obese participants had lower scores than the overweight participants, who themselves reported lower scores than the normal-weight participants for *EPR* (OB/OW mean difference = 0.36, SD = 0.16; OB/NW mean difference = 0.83, SD = 0.1; OW/NW mean difference = 0.47, SD = 0.9). For *RHSC,* the obese and overweight participants reported lower scores than the normal-weight participants (OB/NW mean difference = 0.69, SD = 0.1; OW/NW mean difference = 0.49, SD = 0.8). There were no significant differences between the BMI groups for the *Unconditional Permission to Eat* subscale (*UPE: p* = 0.445).

Concerning the measure of food addiction (*mYFAS*), comparisons were conducted on the symptom count and the symptom severity as well as on the symptom and diagnosis prevalence. The results showed a main effect of BMI groups for the *Symptom Count* (*p <* 0.001) and pairwise comparisons (adjusted for age) highlighted that obese and overweight participants reported higher scores than normal-weight participants (OB/NW mean difference = 0.73, SD = 0.2; OW/NW mean difference = 0.33, SD = 0.1).

Concerning symptom severity, the analyses indicated a main effect for *Loss of control* (*p <* 0.001), *Cut down* (*p <* 0.001), *Time spent* (*p <* 0.05), *Impact activities* (*p <* 0.05), *Withdrawal* (*p <* 0.001), *Tolerance* (*p <* 0.001), *Clinical distress* (*p <* 0.001), and *Clinical impairments* (*p <* 0.001), while the groups did not significantly differ from each other for *Despite problems* (*p* = 0.840). The pairwise comparisons (see Table 2) indicated that the obese participants differed significantly from the normal-weight participants for all the symptoms’ severity except for *Time Spent* (*p* = 0.078). In addition, the obese participants differed significantly from the overweight participants for *Impact activities* (OB/OW mean difference = 0.16, SD = 0.7), *Withdrawal* (OB/OW mean difference = 0.17, SD = 0.1), *Clinical distress* (OB/OW mean difference = 0.28, SD = 0.1), and *Clinical impairments* (OB/OW mean difference = 0.25, SD = 0.1), but these two groups did not differ significantly for *Loss of control, Cut down, Time spent*, and *Tolerance* symptoms’ severity.

**Figure 1:**
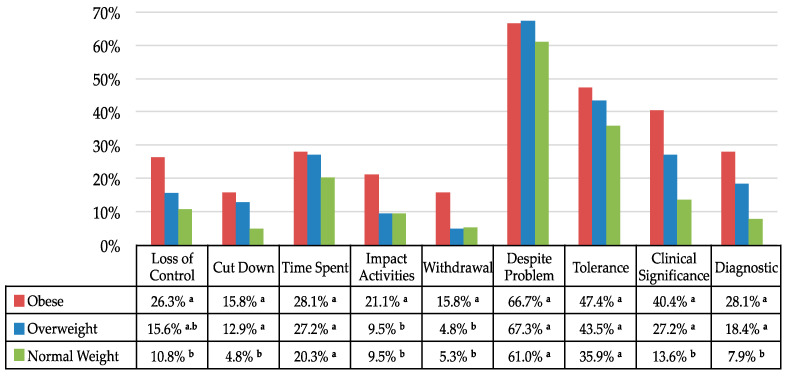
Prevalence of modified Yale Food Addiction Scale (*mYFAS*) symptoms and food addiction diagnosis by BMI group. For each pair of BMI groups, the proportions are compared using a z-test. If a pair of values is significantly different, the values have different subscript letters assigned to them.

Regarding symptoms’ prevalence (see Figure 1), significant differences between the BMI groups emerged for *Loss of control* (χ^2^ (2, *n* = 1142) = 14.0; *p <* 0.001; Cramers’V = 0.11), with a higher proportion among the obese group than the normal-weight group only. The results also showed a main effect for *Impact activities* (χ^2^ (2, *n* = 1142) = 8.0; *p <* 0.05; Cramers’V = 0.08) and *Withdrawal* (χ^2^ (2, *n* = 1142) = 11.1; *p <* 0.005; Cramers’V = 0.10) with a higher proportion among the obese group than among both the overweight and normal-weight groups. In addition, the results highlighted significant differences between the BMI groups for *Cut down* (χ^2^ (2, *n* = 1142) = 22.9; *p <* 0.001; Cramers’V = 0.14), and *Clinical significance* (χ^2^ (2, *n* = 1142) = 41.1; *p <* 0.001; Cramers’V = 0.19), with a higher proportion among both the obese and overweight groups than the normal-weight group. There was no significant main effect of BMI groups for *Time spent* (χ^2^ (2, *n* = 1142) = 5.6; *p* = 0.800), *Despite problems* (χ^2^ (2, *n* = 1142) = 2.7; *p* = 0.259) and *Tolerance* (χ^2^ (2, *n* = 1142) = 5.7; *p* = 0.570).

Finally, the analyses indicated a main effect of BMI groups for the mYFAS *Diagnosis* prevalence (χ^2^ (2, *n* = 1142) = 35.9; *p <* 0.001; Cramers’V = 0.18) and binary logistic regressions showed that relative to the normal-weight group, the odds ratio of meeting the FA diagnosis was 4.56 (95% CI (2.44–8.51), *p <* 0.001; Cramers’V = 0.16) for the obese and 2.63 (95% CI (1.62–4.25), *p <* 0.001; Cramers’V = 0.12) for the overweight participants.

### 3.3. Correlograms

**Figure 2:**
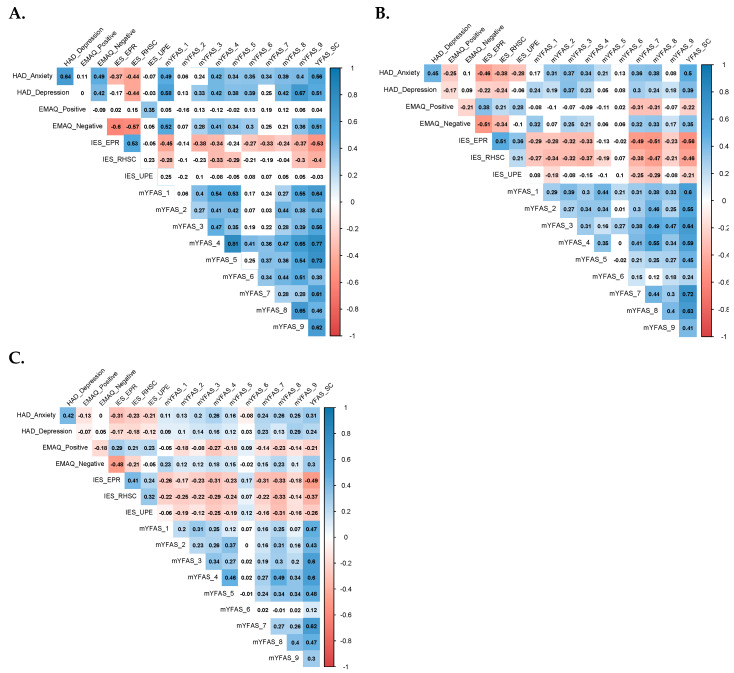
Correlograms for each BMI group. (**A**), Correlogram for Obese group (*n* = 57). (**B**), Correlogram for Overweight group (*n* = 147). (**C**), Correlogram for Normal Weight group (*n* = 938). Positive correlations are displayed in blue and negative correlations are displayed in red. The darkness of the color is proportional to the correlation coefficient, such that the strong correlations (i.e., the closest to −1 or 1) are represented in dark boxes. Nonsignificant correlations are displayed in white. HAD Anxiety: Hospital Anxiety and Depression Scale—Anxiety subscale. HAD Depression: Hospital Anxiety and Depression Scale—Depression subscale. EMAQ Positive: Emotional Appetite Questionnaire Positive subscale. EMAQ Negative: Emotional Appetite Questionnaire Negative subscale. IES-2: Intuitive Eating Scale 2; -EPR: Eating for physical rather than emotional reasons; -RHSC: Reliance on Hunger and Satiety Cues; -UPE: Unconditional Permission to Eat. mYFAS_1: Loss of control; mYFAS_2: Cut down; mYFAS_3: Time spent; mYFAS_4: Impact activities; mYFAS_5: Withdrawal; mYFAS_6: Despite problems; mYFAS_7: Tolerance; mYFAS_8: Clinical distress; mYFAS_9: Clinical impairments.

Figure 2 presents the correlograms of the correlation matrix between the variables of interest for the obese, overweight, and normal-weight groups separately. The results showed that *HAD Anxiety* and *Depression* scores were significantly positively correlated with the majority of mYFAS symptoms severity among all BMI groups. However, mood and symptom severity scores were more strongly correlated among the obese group than among the other two groups, particularly for *Loss of control* (i.e., *mYFAS_1*) and *Clinical impairments* (i.e., *YFAS_9*), with coefficient values around 0.5 for *HAD Anxiety* and 0.6 for *HAD Depression*.

Unlike the findings among the overweight or normal-weight groups, *EMAQ Negative* scores were significantly positively correlated with the *HAD Anxiety* (*r* = 0.49) and *HAD Depression* scores (*r* = 0.42) among the obese group. Regarding intuitive eating (IES-2), the *Reliance on Hunger and Satiety Cues* (*RHSC*) subscale scores were significantly and negatively correlated with *HAD Anxiety*, *HAD Depression*, and *EMAQ Negative* scores among all BMI groups, but the correlation values were the highest among the obese group (*IES-2 RHSC* and *HAD Anxiety* or *HAD Depression*: *r* = −0.44; *IES-2RHSC* and *EMAQ Negative*: *r* = −0.57).

### 3.4. Mediation Analyses

Based on these results, we tested if negative emotional eating (*EMAQ Negative* scores) in the obese group mediated the observed positive association between psychological distress (*HAD Anxiety* and *Depression* scores) and the lack of reliance on internal cues to regulate food intake (*IES-2 RHSC* scores). For each mediation model, path estimates, indirect and total effect estimates, as well as the percentage of mediation, are presented in Table 3.

For both *HAD* subscales, high scores were associated with low *IES-2 RHSC* scores (Model 1: *HAD Dep* → *IES-2 RHSC*; Model 2: *HAD Anx* → *IES-2 RHSC*) and high *EMAQ Negative* scores predicted low *IES-2 RHSC* scores independently from *HAD* scores (Model 1: *EMAQ Neg* → *IES-2 RHSC*; Model 2: *EMAQ Neg* → *IES-2 RHSC*). Moreover, for both models, the indirect effects were significant (Model 1: *HAD Dep* → *EMAQ Neg* → *IES-2 RHSC*; Model 2: *HAD Anx* → *EMAQ Neg* → *IES-2 RHSC*), indicating that for both models *EMAQ Negative* scores did act as mediators in the association between high *HAD Depression* or *Anxiety* scores and low *IES-2 RHSC* scores. The proportion of the total effect explained by the indirect effect was 47.6% and 54.7% for Models 1 and 2, respectively. 

## 4. Discussion

We examined the extent to which obese people differ in their emotionally driven and addictive-like eating behaviors not only from normal-weight but also overweight people in a sample from the French general population. We confirmed previous findings that have been reported in high BMI population, by showing that the two high BMI groups reported higher levels of depressed mood, eating less intuitively but more in response to their negative emotions, and that they presented more severe and/or frequent symptoms of addictive-like eating behaviors than normal-weight people [34,54,55,56]. In addition, we found an increase in FA diagnosis prevalence (as defined by the mYFAS), with the odds for presenting the condition being more than four times higher among the obese group and more than two times higher among the overweight group than among the normal-weight people. The prevalence of FA diagnosis in the obese participants was comparable to the prevalence of FA diagnosis reported in studies using the longer version of the scale (i.e., the YFAS: 15–25% [57]). In all BMI groups, the most often endorsed symptom by the participants was «*Use despite aversive emotional/physical problem*», with comparable high prevalence in the three groups (on average 65%). Although this symptom is commonly reported [33,57], this high rate among the normal-weight group was unexpected as it is much closer to the rates described in clinical samples e.g., bariatric surgery candidates, binge eating disorder: 40-75% [58,59]) than in community samples (9-23% [47,59,60]) using the YFAS and YFAS 2.0. 

Further, we found an increased frequency of the *Loss of control* and *Inability to Cut Down* symptoms by weight classes, but with comparable prevalence between the Obese and Overweight participants. They are both core components and characteristic behavioral features of addiction that have been critically incriminated in the « downwardly escalating dimension » along the continuum of overeating in C. Davis’ psychobiological model of eating behaviors [27]. Interestingly, the same pattern of association between indicators of anxiety or depression, FA, and a lack of intuitive eating was found across all weight classes, suggesting that addictive-like eating may represent a unique phenotype of problematic eating behavior that is not synonymous with BMI and obesity, including a complex pattern of interaction between psychological distress, emotion regulation and addictive process. Such findings suggest that individuals prone to FA may turn to excessive food consumption as a coping strategy for heightened emotional distress, similar to individuals with a substance use disorder [23].

Moreover, besides these findings, we believe the present study also adds to the field by providing a more fine-grained distinction between Obese and Overweight people and highlighting individual characteristics that appeared more specifically associated with the obese phenotype. Indeed, Obese participants reported more severe depressive symptoms than the Overweight participants, which is in line with the well-known depression-obesity association and co-occurrence [54]. Combined with the fact that Obese individuals also reported eating even more than the Overweight participants when facing negative emotions or situations, our study further supports the suggestion of a bidirectional link between obesity and depression, more particularly, with the atypical depression subtype [54,61,62]. Emotional eating has been shown to be (i) exacerbated in obese women, (ii) associated with both consumption of highly palatable food and weight gain [9,55] and (iii) it is a negative factor for post-bariatric surgery weight management outcomes [63]. Moreover, an emerging line of evidence points out that negative EE acts as a mediator between depression and obesity and that it may be a marker of atypical depression [28,31,32]. Here, we found a mediation effect of negative EE on the association between psychological distress (for both depression and anxiety) and the difficulties to rely on hunger and satiety cues, difficulties that are, in turn, known to place the person at risk for increased weight [56]. The present data, thus, complement these observations and suggest that obese individuals get caught in a downward spiral and vicious circle leading to an ‘interoceptive blindness’ due to a specific interplay between their negative affect and their eating patterns. Of important note, it seems this dynamic is not so much an issue of the perceived intensity of the negative affective states as an issue of the obese individual’s negative emotional experience per se, because the Obese group admittedly reported higher levels of depressed mood, but similar levels of anxiety, than the other two groups. Our results are in line with previous studies in non-clinical [7] and clinical samples with obesity or eating disorders [36,64] and point out the role of emotion regulation on eating behavior across different weight classes. While the present findings suggest higher alterations in emotional regulation among individuals with obesity, our study also highlights the role of EE in depression and altered interoception of satiety signals, that is a well-known crucial component for regulating food intake. Our study adds a piece of knowledge on this topic, by showing that individuals with obesity could be more vulnerable to such effects, and offers interesting perspectives for improving intervention approaches aimed at reducing compulsive eating behaviors and body weight. These results also seem to support the *Emotionally Driven Eating Model* [65] considering alterations in emotional regulation and cognitive processing as a key mechanism of inappropriate eating behaviors and overeating. Further studies should address in daily life emotion trajectories, emotional regulation strategies, satiety signals and eating behaviors using Ecological Momentary Assessment to confirm the real time temporal dynamics and relationships between these variables among obese patients.

Further, in addition to replicating the observed association between FA, EE and depression, the present study is, to the best of our knowledge, the first one to statistically compare if the prevalence and severity of FA symptoms vary across high BMI classes. Besides the finding that Obese participants reported more severe levels of *Clinical distress* and *Impairments* than the Overweight participants, *Impact Activities* and *Withdrawal* were found to distinguish these two groups as well. In the mYFAS, the wording of the symptom *Impact activities* clearly refers to the negative emotional experience associated with the overconsumption (i.e., « *I have spent time dealing with negative feelings from overeating certain food*») and the fact that it is frequently endorsed by the obese group is consistent with their high levels of depression. This symptom may be related to ruminative thinking, which is a cognitive process that has been associated with the severity of eating disorders symptomatology in both clinical and non-clinical populations [66] and may lead to EE [67]. Moreover, ruminative thinking has been found to impair cognitive flexibility and decision making, which are processes that have been found to be impaired in obese individuals [6,68]. Additional studies are needed to confirm our suggestion and provide further arguments for incorporating anti-rumination therapy for people with comorbid obesity and depression.

The prevalence of *Withdrawal* symptom was three times higher in the Obese group than the Overweight group. Although the suggestion that withdrawal syndromes occur to certain food items has been subject to heavy criticism in the early days of the FA construct, a growing line of experimental evidence has emerged in animal and human studies, and showing notably psychological signs of withdrawal in humans [69]. The mYFAS was based on DSM-IV-TR criteria of the SUD, so it does not evaluate *Craving,* a symptom that is tightly associated with *Withdrawal.* Therefore, we could not ascertain if its absence biased the results. Nonetheless, the frequency of withdrawal symptom endorsement remains high in obese people even when items on *Craving* are considered using the DMS-5 version of the scale (i.e YFAS 2.0 [47,59]). To gain knowledge on this issue, a recently developed self-report, the Highly Processed Withdrawal Food Scales [70], might prove beneficial in future research. 

Although the current study provides important information about emotionally-driven and addictive-like eating behaviors by weight class, some limitations should be considered. First, researchers should know that women are more prone than men to (i) show symptoms of psychological distress, (ii) report EE, and (iii) to be affected by obesity [15,61]. Therefore, the number of women in our sample could have influenced our results. Another limitation concerns the use of self-reports that raises the question of the ability for introspection, the gap between the participant’s perceptions and realities, or the social desirability bias in the areas of weight and eating behaviors. Furthermore, although some authors highlighted the role of the nutritional and/or chemical composition of HP food in emotionally-driven and addictive-like eating behaviors [71], the type of food consumed was not considered in this study. Finally, personal and psychiatric risk factors for EE, FA and obesity, such as traumatic experiences/PTSD or binge eating disorder [58,72], were not assessed in the study, and these factors may have affected the findings.

Despite these limitations, the present study has important clinical implications. The hypothesis that a distinct mechanism drives excessive weight gain among obese individuals involving EE, psychological distress, and intuitive eating points to the need for specific and integrated interventions in this population. In view of the high level of clinically significant impairments and distress of FA among obese participants, assessment of symptoms and/or diagnosis of food addiction should be systematically considered in this population. A more comprehensive approach integrating emotional dysregulation and addictive-like eating behaviors could improve weight management and quality of life. The key role of EE in this group highlights the need to promote emotion regulation skills in the treatment of obesity. The efficacy of such interventions should be further investigated in randomized controlled trials. 

This study confirms a complex pattern of interaction between psychological distress, emotion regulation and addictive process. Such findings suggest that individuals prone to FA may turn to excessive food consumption as a coping strategy to relieve negative affects, similar to individuals with a substance use disorder. More importantly, this study showed that for the obese individuals emotional eating plays a mediation effect between psychological distress and the difficulties to rely on hunger and satiety cues. This emphasizes the role of emotional dysregulation in obesity risk and addiction vulnerability with a potential significant impact on the perception of satiety signals. In summary, this study highlighted the central role of emotional eating and negative affectivity in the maintenance of non-homeostatic eating behaviors among obese individuals. By showing a specific pathway between psychological distress, emotional eating, and a lack of intuitive eating in obese people, our findings support the hypothesis of a distinct mechanism buffering weight management in this population. It also paves the way for designing interventions that aim to reduce compulsive eating behaviors or body weight in this population. In view of the food addiction prevalence and symptoms’ severity among the obese people, this study suggests that therapeutic approaches of addictive disorders should be proposed in the presence of FA. To progress in this domain, Ecological Momentary Assessments and mobile applications could offer a paradigm shift, first in the way ecologically valid data can be collected in daily life, and then, in turn, in the way personalized care could be offered depending on the individual’s needs.

## Figures and Tables

**Table 1:** Descriptive statistics of the sample.

	*n*	*%*	
**Gender**			
Men	286	25	
Women	856	85	
**Level of education**			
High School degree	26	2.3	
Bachelor’s degree	760	66.8	
Master’s degree	321	28.2	
Doctorate degree	30	2.6	
**mYFAS**			
Diagnosis	117	10.2	
	*M*	*SD*	*Min–Max*
**Age**	23.4	7.5	18–68
**BMI**	22.7	3.8	18.5–57.8
**HAD**			
Anxiety	8.0	3.8	0–19
Depression	4.1	3.2	0–17
**EMAQ**			
Positive	4.9	0.9	1–8.6
Negative	4.4	1.3	1–8.8
**IES-2**			
EPR	3.3	1.1	1–5
RHSC	3.3	0.9	1–5
UPE	3.5	1.0	1–5
**mYFAS**			
Symptoms Count	1.6	1.4	0–7

M: Mean. SD: Standard Deviation. BMI: Body Mass Index. HAD: Hospital Anxiety and Depression Scale. EMAQ: Emotional Appetite Questionnaire. IES-2: Intuitive Eating Scale 2; EPR: Eating for physical rather than emotional reasons; RHSC: Reliance on Hunger and Satiety Cues; UPE: Unconditional Permission to Eat. mYFAS: modified Yale Food Addiction Scale.

**Table 2:** BMI group comparisons.

	Obese (OB)		Overweight (OW)		Normal Weight (NW)										
*n*	57		147		938										
**%**	5.0		12.9		82.1										
Measure	M	SD	M	SD	M	SD					Pairwise comparisons adjusted for age				
							*F*	*df*	*p*	*η_p_* ^2^	Groups’ comparison	*F*	*df*	*p*	*η_p_* ^2^
**HAD**															
Anxiety	8.8	4.3	8.1	3.9	7.9	3.7	1.9	2.1138	NS	---	---	---	---	---	---
Depression	5.8	4.4	4.2	3.0	3.9	3.1	9.4	2.1138	<0.001	0.02	OB > OW	8.9	1.201	0.003	0.04
											OB > NW	16.4	1.992	<0.001	0.02
											OW = NW	---	---	---	---
**EMAQ**															
Positive	4.3	1.2	4.5	1.0	4.8	0.9	14.3	2.1127	<0.001	0.02	OB = OW	---	---	---	---
											OB < NW	11.6	1.982	<0.001	0.01
											OW < NW	13.6	1.1073	<0.001	0.01
Negative	5.5	1.6	4.9	1.3	4.3	1.2	34.5	2.1131	<0.001	0.06	OB > OW	6.8	1.199	0.01	0.03
											OB > NW	49.9	1.986	<0.001	0.05
											OW > NW	29.7	1.1076	<0.001	0.03
**IES-2**															
EPR	2.6	1.1	2.9	1.0	3.4	1.1	28.1	2.1138	<0.001	0.05	OB < OW	4.5	1.201	0.038	0.02
											OB < NW	35.4	1.992	<0.001	0.03
											OW < NW	27.9	1.1082	<0.001	0.03
RHSC	2.7	1.0	2.9	1.0	3.4	1.0	30.3	2.1138	<0.001	0.05	OB = OW	---	---	---	---
											OB < NW	31.0	1.992	<0.001	0.03
											OW < NW	38.2	1.1082	<0.001	0.03
UPE	3.5	0.9	3.3	0.9	3.5	1.0	2.5	2.1138	NS	---	---	---	---	---	---
**mYFAS Symptom Severity**															
1—Loss of control	0.3	0.4	0.2	0.4	0.1	0.3	5.7	2.1138	<0.001	0.01	OB = OW	---	---	---	---
											OB > NW	17.1	1.989	<0.001	0.02
											OW = NW	---	---	---	---
2—Cut down	0.2	0.4	0.2	0.4	0.1	0.2	10.9	2.1138	<0.001	0.02	OB = OW	---	---	---	---
											OB > NW	12.9	1.992	<0.001	0.01
											OW > NW	14.6	1.1082	0.001	0.01
3—Time spent	0.4	0.7	0.4	0.7	0.2	0.5	4.1	2.1138	<0.05	0.01	OB = OW	---	---	---	---
											OB=NW	---	---	---	---
											OW > NW	6.4	1.1082	0.012	0.01
4—Impact activities	0.3	0.6	0.1	0.4	0.1	0.4	4.2	2.1138	<0.05	0.01	OB > OW	4.3	1.201	0.04	0.02
											OB > NW	8.1	1.992	0.004	0.01
											OW = NW	---	---	---	---
5—Withdrawal	0.2	0.6	0.1	0.3	0.1	0.3	6.6	2.1138	<0.001	0.01	OB > OW	7.1	1.201	0.008	0.03
											OB > NW	12.9	1.992	0.001	0.01
											OW = NW	---	---	---	---
6—Despite problems	1.4	1.4	1.6	1.4	1.5	1.5	0.4	2.1138	NS	---	---	---	---	---	---
7—Tolerance	1.2	1.5	1.0	1.3	0.7	1.2	7.4	2.1138	<0.001	0.01	OB = OW	---	---	---	---
											OB > NW	9.4	1.990	0.002	0.01
											OW > NW	7.6	1.1081	0.006	0.01
8—Clinical distress	0.6	0.9	0.4	0.7	0.2	0.5	27.3	2.1138	<0.001	0.05	OB > OW	6.8	1.200	0.01	0.03
											OB > NW	45.1	1.992	<0.001	0.04
											OW > NW	18.3	1.1082	<0.001	0.02
9—Clinical impairments	0.4	0.8	0.2	0.5	0.1	0.3	20.5	2.1138	<0.001	0.03	OB > OW	7.7	1.200	0.006	0.04
											OB > NW	40.1	1.992	<0.001	0.04
											OW > NW	6.8	1.1082	0.009	0.01
**mYFAS** **Symptom Count**	2.2	1.8	1.8	1.6	1.5	1.4	10.8	2.1138	<0.001	0.02	OB = OW	---	---	---	---
											OB > NW	17.1	1.989	<0.001	0.02
											OW > NW	8.2	1.1079	0.004	0.01

*df:* degrees of freedom. *η_p_*^2^: partial eta-squares. OB: Obese; OW: Overweight; NW: Normal Weight. HAD: Hospital Anxiety and Depression Scale. EMAQ: Emotional Appetite Questionnaire. IES-2: Intuitive Eating Scale 2; EPR: Eating for physical rather than emotional reasons; RHSC: Reliance on Hunger and Satiety Cues; UPE: Unconditional Permission to Eat. mYFAS: modified Yale Food Addiction Scale. NS: not significant.

**Table 3:** Direct, indirect, and total effects of the two mediation models among the obese group.

Models Tested	% Mediation	B	SE	*p*	95% CI	
					Lower	Upper
Model 1: HAD Dep → EMAQ Neg → IES-2 RHSC						
Direct effects	52.4					
Path a: HAD Dep → EMAQ Neg		0.138	0.05	0.004	0.035	0.225
Path b: EMAQ Neg → IES-2 RHSC		−0.309	0.07	<0.001	−0.448	−0.165
Path c: HAD Dep → IES-2 RHSC		−0.047	0.03	0.109	−0.100	0.013
Indirect effect (a X b)	47.6					
HAD Dep → EMAQ Neg → IES-2 RHSC		−0.043	0.018	0.020	−0.082	−0.0096
Total effect (c + a X b)	100					
HAD Dep → IES-2 RHSC + HAD Dep → EMAQ Neg → IES-2 RHSC		−0.089	0.027	<0.001	−0.133	−0.029
Model 2: HAD Anx → EMAQ Neg → IES-2 RHSC						
Direct effects	45.3					
Path a: HAD Anx → EMAQ Neg		0.171	0.04	<0.001	0.085	0.2446
Path b: EMAQ Neg → IES-2 RHSC		−0.303	0.08	<0.001	−0.453	−0.1446
Path c: HAD Anx → IES-2 RHSC		−0.043	0.027	0.116	−0.0966	0.0112
Indirect effect (a X b)	54.7					
HAD Anx → EMAQ Neg → IES-2 RHSC		−0.052	0.018	0.004	−0.089	−0.018
Total effect (c + a X b)	100					
HAD Anx → IES-2 RHSC + HAD Anx → EMAQ Neg → IES-2 RHSC		−0.094	0.023	<0.001	−0.138	−0.047

B: Standardized estimate. SE: Standard Error. 95% CI: 95%Confidence Interval. See supplementary Figure S1 for an illustration of Paths a, b, and c as well as the indirect and total effects.

## Supplementary Materials

The following are available online at https://www.mdpi.com/2072-6643/12/10/2962/s1, Figure S1: Mediation analyses path diagram.
