# Supplementary material for: The Psycho-Affective Roots of Obesity: Results from a French Study in the General Population
Source: Nutrients. 2020 Sep 28;12(10):2962. doi: 10.3390/nu12102962 (PMC7650670; doi:10.3390/nu12102962)
Supplement: Supplementary file 1 [file nutrients-12-02962-s001.pdf]

Supplementary Materials

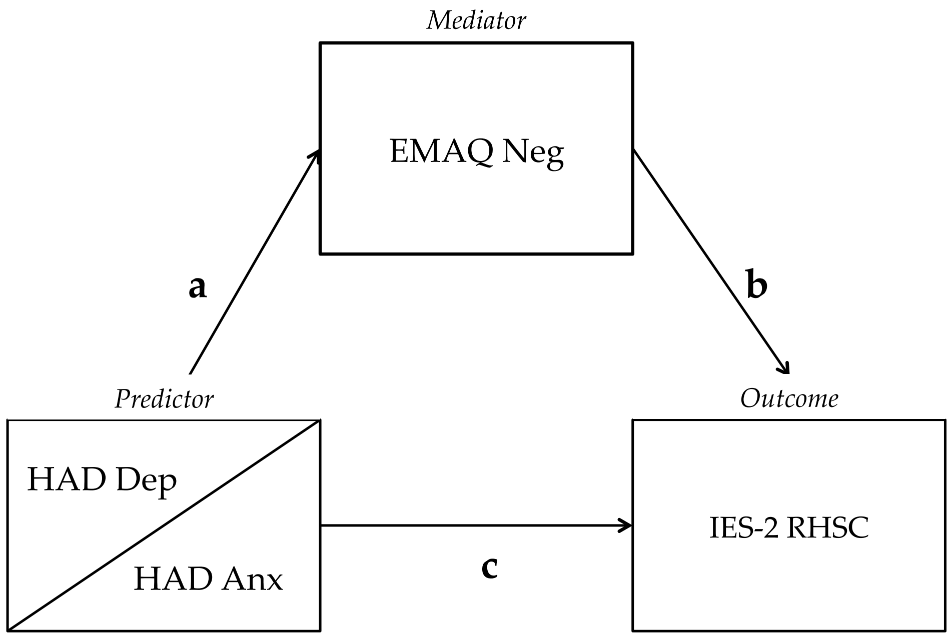

Figure S1. Mediation analyses path diagram
Direct effects : c
Indirect effects : a x b
Total effect : c + a x b
